# S226. A STUDY COMPARING WEIGHT GAIN FROM ALKS 3831 TO OLANZAPINE IN EARLY-ILLNESS YOUNG ADULTS WITH SCHIZOPHRENIFORM, SCHIZOPHRENIA, OR BIPOLAR I DISORDER

**DOI:** 10.1093/schbul/sby018.1013

**Published:** 2018-04-01

**Authors:** Adam Simmons, David McDonnell, Ying Jiang, Christine Graham, Bernard Silverman

**Affiliations:** Alkermes, Inc

## Abstract

**Background:**

Optimal pharmacological efficacy is crucial in first-episode and early-episode schizophrenia and bipolar I disorder. Olanzapine (OLZ) is a highly efficacious antipsychotic indicated for the treatment of schizophrenia and bipolar I disorder. However, the clinical utility of OLZ may be limited by a propensity to cause significant weight gain and increased metabolic side effects, especially for patients early in the course of their illness. ALKS 3831 is composed of a flexible dose of olanzapine (OLZ) and a fixed dose of 10 mg of samidorphan (SAM), formulated as a bilayer tablet. ALKS 3831 has been shown in Phase 1 and Phase 2 studies to result in significantly less weight gain than OLZ while delivering equivalent antipsychotic efficacy. This Phase 3, 12-week study, is designed to evaluate the effect of ALKS 3831 on body weight in young adults early in the course of diagnosis of a serious mental illness, including a schizophreniform, schizophrenia, or bipolar I disorder diagnosis.

**Methods:**

This is an international (Austria, Germany, Ireland, Israel, Italy, Poland, Spain, UK, and USA) two-arm, double-blind, active-comparator–controlled, multicentre study (planned N=250) that started enrollment in 2017. Key inclusion criteria are a primary diagnosis of schizophreniform disorder, schizophrenia, or bipolar I disorder; a body-mass index (BMI) of ≥18.0 and ≤27.0 kg/m2; and meeting specific criteria for duration of illness and prior antipsychotic exposure. Patients with a bipolar I diagnosis must be in the manic phase. In the US sites, men and women must be aged ≥16 to <40 years at screening, and in Europe, aged ≥18 to <40 years. Exclusion criteria include diagnosis of additional psychiatric conditions and use of prohibited drugs.

**Results:**

Patients will be randomised 1:1 to receive either OLZ or ALKS 3831 treatment for 12 weeks. ALKS 3831 (OLZ + SAM) and matched OLZ + placebo (OLZ + PBO) will be provided as bilayer tablets to be taken by mouth once daily and doses will include ALKS 3831 (OLZ/SAM) 5/10 mg, 10/10 mg, 15/10 mg, 20/10 mg, or (OLZ/PBO) 5 mg, 10 mg, 15 mg, or 20 mg. The primary endpoint will be percent change in body weight from baseline to Week 12. Secondary and exploratory endpoints will include the proportion of patients who gain ≥7% and ≥10% of baseline body weight, metabolic parameters (change in fasting lipids and glucose), body composition measured through bioimpedance, clinical global impression, and safety, pharmacokinetic, and pharmacodynamic parameters. Patients will be offered a supportive clinical care programme during the 12-week treatment period, and also receive a daily medication-adherence monitoring and reminder system (via smartphones).

**Discussion:**

Patients who complete the study will have the option to participate in an open-label 2-year extension of ALKS 3831 (based on clinician and patient decision).

EudraCT number: 2017-000497-11; ClinicalTrials.gov identifier: NCT03187769

